# Emergency radiological examination of the externally stabilized pelvis – there is a catch to it: lessons learned from two cases with symphyseal disruption despite initial inconspicuous computed tomography

**DOI:** 10.1186/s12893-016-0126-5

**Published:** 2016-03-12

**Authors:** Jörg Bayer, Thorsten Hammer, Dirk Maier, Norbert Paul Südkamp, Oliver Hauschild

**Affiliations:** Department of Orthopedics and Trauma Surgery, Medical Center - University of Freiburg, Hugstetter Str. 55, 79106 Freiburg, Germany; University Emergency Center, Medical Center - University of Freiburg, Hugstetter Str. 55, 79106 Freiburg, Germany

**Keywords:** Pelvic injury, Pelvic fracture, Symphyseal disruption, Pelvic stabilization, Pelvic binder, Computed tomography, Missed injury, Patient safety

## Abstract

**Background:**

Preclinical and early clinical external pelvic stabilization using commercially available devices has become common in trauma patient care. Thus, in the emergency department an increasing number of patients will undergo radiographic evaluation of the externally stabilized pelvis to exclude injuries. While reports exist where injuries to the pelvis were elusive to radiological examination due to the pelvic immobilization we elaborate on an algorithm to remove an external pelvic stabilizing device, prevent delayed diagnosis of pelvic disruption and thus increase patient safety.

**Case presentation:**

We report on two patients with external pelvic stabilization presenting with an inconspicuous pubic symphysis on initial pelvic computed tomography scans. The first patient was an otherwise healthy 51-year old male being run over by his own car. He received external pelvic stabilization in the emergency department. The second patient was a 36-year old male falling from a ladder. In this patient external pelvic stabilization was performed at the scene. In the first patient no pelvic injury was obvious on computed tomography. In the second patient pelvic fractures were diagnosed, yet the presentation of the pubic symphysis appeared normal. Nevertheless, complete symphyseal disruption was diagnosed in both of them upon removal of the external pelvic stabilization and consequently required internal fixation.

**Conclusion:**

Based on our experience we propose an algorithm to “clear the initially immobilized pelvis” in an effort to minimize the risk of missing a serious pelvic injury and increase patient safety. This is of significant importance to orthopedic trauma surgeons and emergency physicians taking care of injured patients.

## Background

Pelvic fractures are markers for considerable energy transfer during accident and severity of injury, thus they are one of the potentially life-threatening injuries identified during the primary assessment of patients with major trauma [1]. Epidemiologically, pelvic fractures represent up to 6 % of all fractures in adults and occur in one fifth of all polytrauma cases [2]. Unstable pelvic fractures occur in up to 20 % of pelvic fractures and mortality can exceed 20 % in complex pelvic injury [3]. A study by Evans et al. showed that 23 % of deaths due to hemorrhage following trauma were due to bleeding from pelvic fractures [4]. Since bleeding often occurs from cancellous bone surfaces, the presacral venous plexus and iliac vessels in unstable pelvic fractures, reduction and stabilization of pelvic fractures prevents further blood loss by limiting the bleeding from the fracture fragments and reducing pelvic volume [5]. Many tools for external emergency stabilization of the pelvis exist and some of which have been shown to lower the incidence of lethal pelvic bleeding [6]. While previous management principles emphasized the importance of pelvic physical examination to identify tenderness or instability as an indicator of pelvic fracture [7], this doctrine has somewhat changed over time. Recently, in preclinical and early clinical care, recommendations exist for routine external pelvic stabilization (EPS) in unresponsive or hemodynamically unstable trauma patients with clues of pelvic trauma, deliberately omitting physical examination of the pelvis in these cases [1, 8]. In the alert, oriented, cooperative patient with no distracting injury mentioning of present pain in the pelvic area, including the lower back (assessing the sacroiliac joint) and groin should call for routine immobilization of the pelvis [1]. Thus, in our experience, an increasing number of patients are referred to the emergency department with externally stabilized pelvis and will routinely undergo “radiological clearance” for pelvic fractures by pelvic radiography and/or computed tomography scans. We present two cases with an externally stabilized pelvis eluding pubic symphyseal disruption on initial radiological workup and propose an algorithm to safely clear an initially immobilized pelvis.

## Case presentations

### Patient 1

**Fig. 1:**
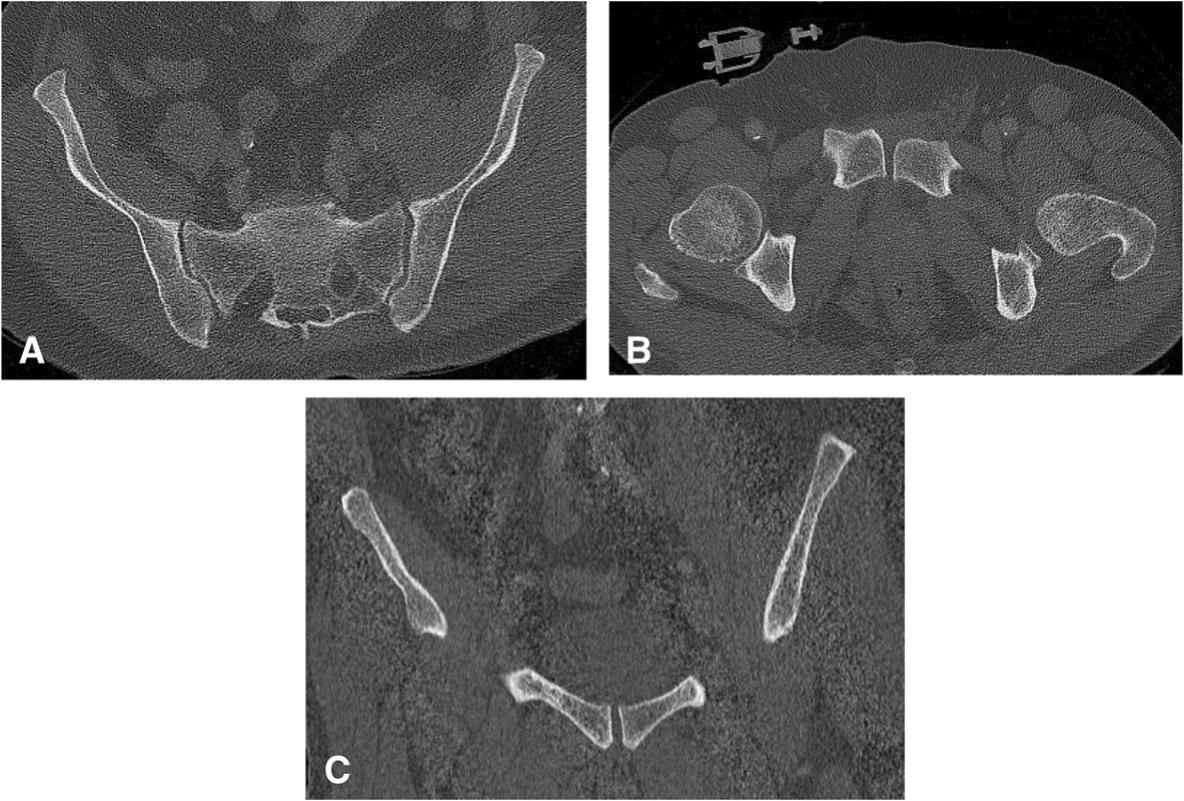
Initial computed tomography scans. CT scans of the pelvis with external pelvic stabilization (SAM Pelvic Sling™ II) in place (**b**). Slightly disproportionate left iliosacral joint, yet no bony injuries are visible (**a**). The pubic symphysis appears normal in width and configuration (**b**, **c**)

**Fig. 2:**
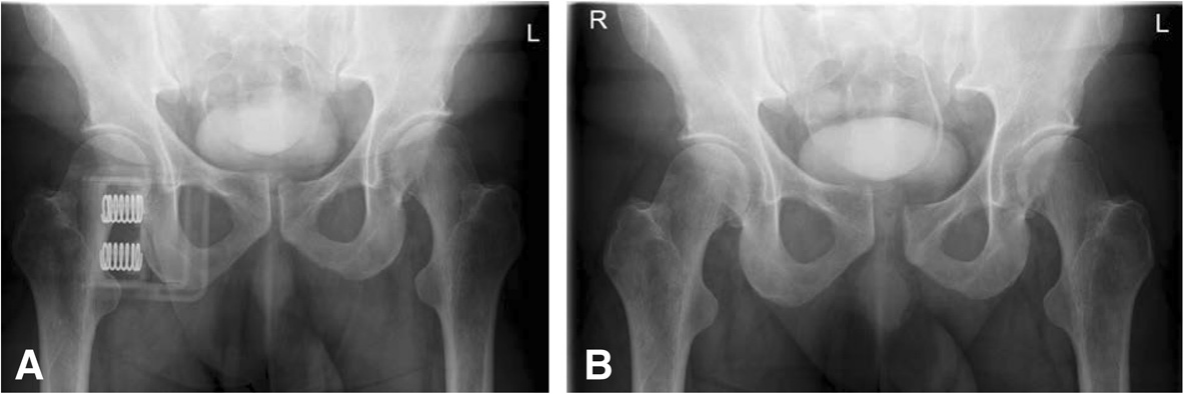
Plain pelvic radiographs after CT scan. Anteroposterior pelvic radiograph with (**a**) and without (**b**) the SAM Pelvic Sling™ II. The symphyseal gap appears normal, yet little vertical displacement of the pubic symphysis can be noted as a subtle hint of unstable pelvic injury (**a**). Without external stabilization the left iliosacral joint and the pubic symphysis are decisively wider (**b**)

**Fig. 3:**
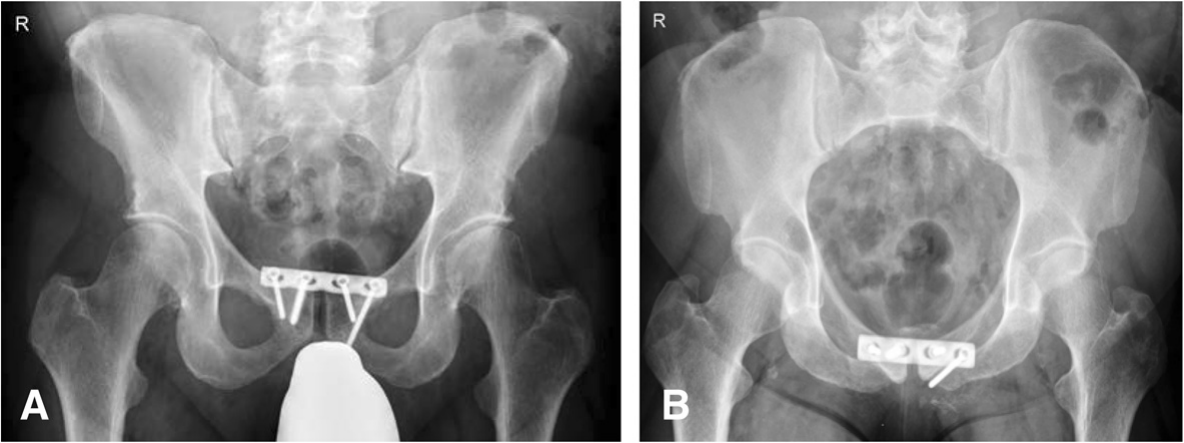
Postoperative plain pelvic radiographs. Anteroposterior (**a**) and inlet (**b**) view. Plating osteosynthesis of the pubic symphysis results in anatomical reduction and stabilization of the pelvic ring

A 51-year-old otherwise healthy male tried to stop a driverless car running down a hill by pushing the brake while trying to get into the car. Unfortunately, the car slanted and finally tipped over, burying the patient mainly at the pelvic level underneath. Emergency medical service (EMS) personal found the freed patient with suspected pelvic injury but otherwise no respiratory or hemodynamic compromise as well as an inconspicuous neurological state. After sufficient intravenous analgesia the patient was air-lifted to our hospital. On admission the patient was awake and alert without any relevant cardiorespiratory dysfunction (oxygen saturation 97 % without oxygen supply, heart rate 86 bpm and blood pressure of 150/95 mmHg). On clinical examination the patient exhibited a hematoma and pain at the left shoulder as well as a tender pelvis, especially at the left posterior region, and the pelvis was suspected to be unstable. Following the institutional protocol, immediate pelvic stabilization was carried out using an external device (SAM Pelvic Sling™ II, SAM Medical Products®, Wilsonville, OR, USA). Due to the mechanism of injury and suspected pelvic injury the patient was subjected to a computed tomography scan (CT). Representative sections of the pelvic CT are shown in Fig. 1. Conferring with the radiologist on call no bony injuries were detected. Apart from a slightly widened left sacroiliac joint no injuries of the visualized joints were seen, either. Because of the clinical findings conventional radiographs were performed with the pelvic stabilizer engaged and released, in order to reveal possible injuries. Surprisingly, without EPS the pubic symphysis was wide open at 2.2 cm, additionally the left sacroiliac joint was significantly wider, too (Fig. 2). Finally, this set of pelvic radiographs proved the non-osseous pelvic disruption (type B 1.1 according to the Tile classification [9]) and the patient was taken to the operating room for operative pelvic stabilization. Ensuing, after internal stabilization of the pubic symphysis (Fig. 3), the patient developed a pneumonia which resolved under antibiotic treatment and the patient was discharged to rehabilitation on the 21^st^ day after his accident.

### Patient 2

**Fig. 4:**
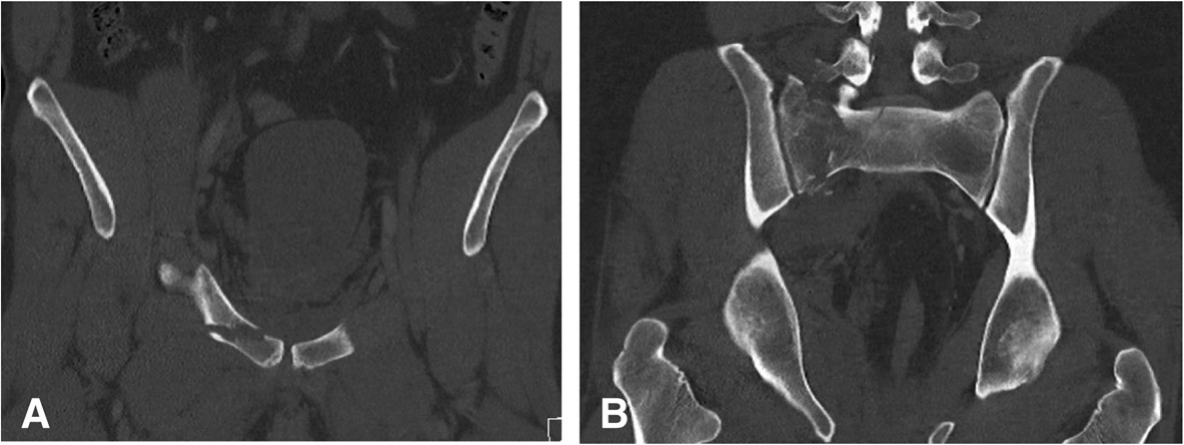
Initial computed tomography scans. Pelvic computed tomography with SAM Pelvic Sling™ II in place. Fracture of the right superior pubic ramus with regular configuration of the pubic symphysis (**a**). Ipsilateral fracture of the sacrum (**b**)

**Fig. 5:**
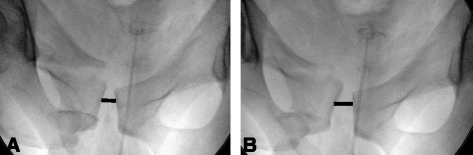
Intraoperative fluoroscopy of the pelvis. Pubic symphysis width has increased without external pelvic stabilization (**b**). With the SAM Pelvic Sling^TM^ II attached pubic symphysis has a regular configuration and the fractured superior pubic ramus is slightly overriding (**a**)

**Fig. 6:**
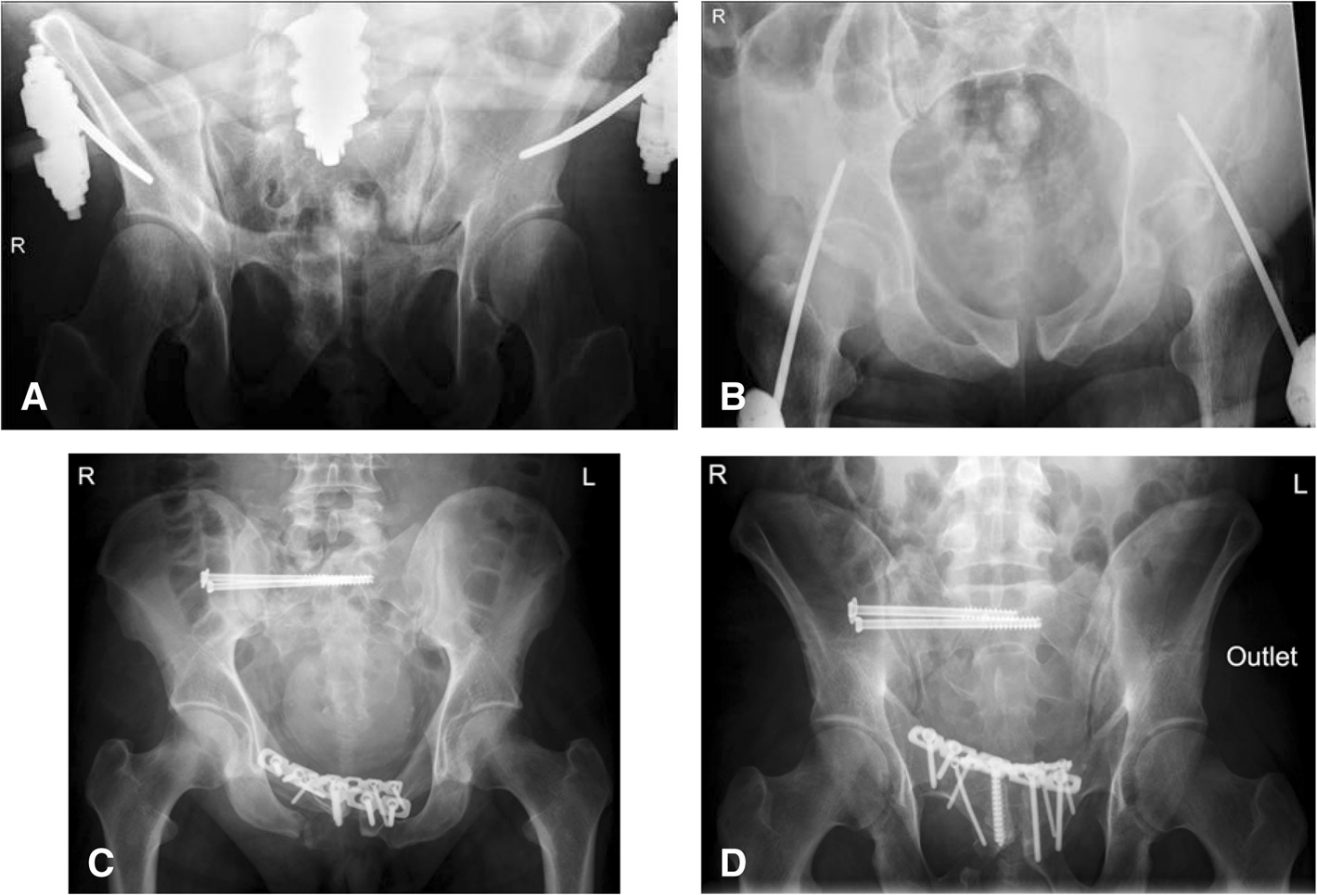
Postoperative plain pelvic radiographs. Temporary fixation of the anterior pelvis with supraacetabular external fixator (**a**, **b**) shows good reduction of the pubic symphysis. After definitive osteosynthesis iliosacral screws and anterior plating stabilize the pelvic ring (**c**, **d**)

A 36-year-old otherwise healthy foreign male was found wincing in the vicinity of a ladder underneath a warehouse roof (height approximately 5 meters). Upon arrival of the EMS doctor the patient was able to maintain an open airway, the chest was stable und bilateral breath sounds were present. The patient was hemodynamically stable. The vitals were: respiratory rate 18/min, oxygen saturation 100 % without oxygen supply, heart rate 95 bpm, blood pressure 180/90 mmHg, blood sugar 135 mg/dl. Because of pelvic pain and suspected pelvic instability EPS was performed with the SAM Pelvic Sling^TM^ II (SAM Medical Products®, Wilsonville, OR, USA). The Glasgow Coma Scale (GCS) was 14 (E4/V4/M6) and a fracture of the left lower leg was clinically diagnosed. After rapid sequence induction the patient was endotracheally intubated and a total crystalloid volume of 2000 ml was administered en route to our hospital. On admission we saw an intubated and mechanically ventilated patient. The vitals were: respiratory rate 12/min, etCO_2_ 36 mmHg, oxygen saturation 100 % with FiO_2_ 1.0, heart rate 80 bpm, regular sinus rhythm, blood pressure 150/90 mmHg. On physical examination no injuries to the cranium, chest or abdominal cavity were clinically evident. Sonography (Focused Assessment with Sonography in Trauma (FAST)) did not reveal any fluid collections and the pelvis had been immobilized as described above. The left lower leg was evidently fractured on clinical examination. The patient underwent whole-body CT that revealed bilateral lung contusions, a fracture of the right transverse process of the 5^th^ lumbar vertebra, a right sided sacral fracture and superior and inferior pubic rami fractures (Fig. 4). The patient was taken to the operating room for supraacetabular external fixation of the pelvis and external fixation of the left lower leg. Upon arrival in the operating room provisional EPS was removed. Astonishingly, intraoperative fluoroscopy of the pelvis revealed a significantly wider pubic symphysis, suggestive of symphyseal disruption (Fig. 5). In due course, internal fixation of the anterior pelvis was performed together with iliosacral screw fixation. Intraoperative complete symphyseal disruption was confirmed warranting extended plate osteosynthesis of the pubic symphysis (Fig. 6). After converting the external fixation of the left lower leg to intramedullary nailing the further course of the patient was uneventful and the patient was repatriated on day eight after trauma.

## Discussion

Preclinical and early clinical management of trauma patients may involve EPS without physical examination of the pelvis, depending on vital signs and located pain as well as trauma kinematics, only [1, 8]. In our cases pre- as well as early clinical pelvic immobilization was deemed necessary because of pelvic pain and/or trauma kinematics. Upon arrival at the hospital both patients were treated according to the basic ATLS protocol [8]. Whether plain pelvic radiography should precede further diagnostics in suspected pelvic trauma is debated, especially if computed tomography is planned [10]. In our cases whole-body computed tomography was deemed indicated because of the trauma kinematics and suspected injuries by the receiving orthopedic surgeon, thus initial plain radiographs were omitted.

For plain pelvic radiographs, sensitivity and specificity in relation to CT has been found to be 67 and 100 % [10], respectively, whereas computed tomography of the pelvis is reported to provide a sensitivity and specificity of almost 100 % in diagnosis of pelvic injury [11]. Nevertheless, in an accurately externally stabilized pelvis symphyseal and sacroiliac-joint approximation is achieved [12, 13] and reports exist where injuries to the pelvis weren’t obvious due to the pelvic immobilization [13, 14]. Yet, in these previous reports either the symphyseal and sacroiliac joint disruption was documented on x-rays obtained prior to external pelvic stabilization and CT [13] or a fracture of the sacrum was associated with a symphyseal disruption [14]. As opposed to these previous publications we report on a solely non-osseous pelvic injury which was nearly missed on initial pelvic CT due to EPS and a symphyseal disruption undetected in pelvic CT scans combined with sacral and pubic rami fractures.

## Conclusion

Based on our experience with symphyseal injuries masked by EPS we recommend leaving the device engaged during the initial assessment. In case of a radiologically detected pelvic fracture mandating immediate surgical stabilization (e.g. external pelvic fixator) we recommend leaving the device in place until operative intervention, but to control for symphyseal injuries using intraoperative fluoroscopy. In the remaining cases we recommend to distinguish between the fully alert patient and others. In intubated, sedated or otherwise obtunded patients we would remove the EPS and afterwards obtain an anteroposterior pelvic x-ray to check for sacroiliac-joint and symphyseal widening. Only in alert, oriented and cooperative patients with no distracting injury and absence of pain in the pelvic area under physical examination, we would remove the pelvic stabilization and abstain from further pelvic imaging.

## Consent

Written informed consent was obtained from both patients for publication of this case report and any accompanying images. A copy of the written consent is available for review by the Editor-in-Chief of this journal. The case presentations have been approved by our institutional ethics committee (EK 277/15).

## Abbreviations

ATLS: advanced trauma life support
CT: computed tomography scan
EMS: emergency medical service
EPS: external pelvic stabilization
FAST: focused assessment with sonography in trauma
GCS: Glasgow Coma Scale
